# Dr. Corson’s New Physical Signs

**Published:** 1859-09

**Authors:** 


Dr. Corson's New Physical Signs.—In a notice of Dr. Corson's "Paper on the Management of the Shoulders in Examinations of the Chest," we alluded to a new physical sign of disease of the lungs described by him, the diminution of motion in the chest on the affected side.—We have had opportunites of confirming the value of this sign, and have found it so striking that it is difficult to explain how it has so long escaped observation. In two patients now under our care, each of whom has extensive tuberculous infiltration of the left lung, the difference between the motion of the two sides is remarkable. Viewed from behind the patient's right shoulder is seen to rise and fall with each respiratory act, while the left shoulder is almost motionless. In each case we successfully predicted that the disease would be found on the left side. We have not yet had occasion to notice Dr. Corson's other new sign, the "moist respiration," but we would again call attention to this, as well as to the other valuable practical suggestions contained in his paper, which shows him to be a man of close observation.—*Ib.*

